# Impact of Maternal HIV Infection and Placental Malaria on the Transplacental Transfer of Influenza Antibodies in Mother–Infant Pairs in Malawi, 2013–2014

**DOI:** 10.1093/ofid/ofz383

**Published:** 2019-08-28

**Authors:** Antonia Ho, Gugulethu Mapurisa, Mwayiwawo Madanitsa, Linda Kalilani-Phiri, Steve Kamiza, B Makanani, Feiko O Ter Kuile, Amelia Buys, Florette Treurnicht, Dean Everett, Victor Mwapasa, Marc-Alain Widdowson, Meredith Mcmorrow, Robert S Heyderman

**Affiliations:** 1 MRC-University of Glasgow Centre for Virus Research, Glasgow, UK; 2 Malawi-Liverpool-Wellcome Trust Clinical Research Programme, Blantyre, Malawi; 3 Institute of Infection and Global Health, University of Liverpool, Liverpool, UK; 4 University of Malawi College of Medicine, Blantyre, Malawi; 5 Department of Obstetrics, Queen Elizabeth Central Hospital, Blantyre, Malawi; 6 Liverpool School of Tropical Medicine, Pembroke Place, Liverpool, UK; 7 Centre for Respiratory Diseases and Meningitis, National Institute for Communicable Diseases of the National Health Laboratory Service, Sandringham, South Africa; 8 Department of Medical Virology, School of Pathology, Faculty of Health Sciences, University of the Witwatersrand, Johannesburg, South Africa; 9 MRC Centre for Inflammation Research, University of Edinburgh, Edinburgh, UK; 10 Division of Global Health Protection, CDC Kenya, Kenya; 11 Influenza Division, US Centers for Disease Control and Prevention, Atlanta, Georgia; 12 Division of Infection and Immunity, University College London, London, UK

**Keywords:** influenza, HIV, malaria, antibodies, transplacental transfer

## Abstract

**Background:**

Maternal influenza vaccination protects infants against influenza virus infection. Impaired transplacental transfer of influenza antibodies may reduce this protection.

**Methods:**

We conducted a cross-sectional study of influenza vaccine–naïve pregnant women recruited at delivery from Blantyre (urban, low malaria transmission) and Chikwawa (rural, high malaria transmission) in Southern Malawi. HIV-infected mothers were excluded in Chikwawa. Maternal and cord blood antibodies against circulating influenza strains A/California/7/2009, A/Victoria/361/2011, B/Brisbane/60/2008, and B/Wisconsin/1/2010 were measured by hemagglutination inhibition (HAI). We studied the impact of maternal HIV infection and placental malaria on influenza antibody levels in mother–infant pairs in Blantyre and Chikwawa, respectively.

**Results:**

We included 454 mother–infant pairs (Blantyre, n = 253; Chikwawa, n = 201). HIV-infected mothers and their infants had lower seropositivity (HAI titer ≥1:40) against influenza A(H1N1)pdm09 (mothers, 24.3 vs 45.4%; *P* = .02; infants, 24.3 vs 50.5%; *P* = .003) and A(H3N2) (mothers, 37.8% vs 63.9%; *P* = .003; infants, 43.2 vs 64.8%; *P* = .01), whereas placental malaria had an inconsistent effect on maternal and infant seropositivity. In multivariable analyses, maternal HIV infection was associated with reduced infant seropositivity (A(H1N1)pdm09: adjusted odds ratio [aOR], 0.34; 95% confidence interval [CI], 0.15–0.79; A(H3N2): aOR, 0.43; 95% CI, 0.21–0.89). Transplacental transfer was not impaired by maternal HIV or placental malaria.

**Conclusions:**

Maternal HIV infection influenced maternal antibody response to influenza A virus infection, and thereby antibody levels in newborns, but did not affect transplacental antibody transfer.

Pregnant women and infants under 6 months are at increased risk of severe influenza complications [1]. During the 2009 influenza A(H1N1) pandemic, pregnant women had higher rates of hospital and intensive care admissions [2, 3], as well as a 5-fold higher risk of death [4], compared with nongravid women. Some studies of pregnancy outcomes have suggested increased an risk of pregnancy complications attributable to maternal influenza illness; others have not [5]. The World Health Organization (WHO) designated pregnant women as the highest priority for influenza vaccination [6], though few low- and middle-income countries have vaccination programs targeted at pregnant women [7].

Recent randomized trials have shown that influenza vaccination in pregnancy not only protects the mother but also protects young infants against influenza and its complications [8, 9]. This is partly due to the reduced risk of maternal infection and transmission to the infant and partly due to active transplacental transfer of maternal antibodies generated from immunization [10]. The latter is crucial for infants <6 months, who have higher rates of influenza-associated hospitalization [11] but are ineligible to receive influenza vaccines [12].

HIV infection and malaria are prevalent in sub-Saharan Africa; maternal HIV infection and placental malaria have been associated with impaired transplacental transfer of maternal antibodies against a number of vaccine-preventable diseases including tetanus, measles, and *Streptococcus pneumoniae* [13–16]. HIV-infected pregnant women are an important target group for influenza vaccination, as HIV-infected adults [17] and pregnant women [18] have greater risk of severe influenza, whereas the impact of malaria co-infection is poorly studied [19]. Before implementing maternal influenza vaccination in this region, the effects of maternal HIV and placental malaria on maternal and newborn humoral immunity against influenza, as well as their potential impact on the efficacy of antenatal influenza vaccination, require evaluation. A randomized trial of inactivated influenza vaccine in pregnant women found lower seroconversion to all vaccine strains in HIV-infected mothers, compared with HIV-uninfected mothers, but the efficiency of transplacental antibody transfer was similar [20]. No studies to date have evaluated the effect of placental malaria on maternal–fetal transfer of influenza antibodies.

We investigated the effect of maternal HIV infection and placental malaria on influenza antibody levels in unvaccinated pregnant women and their newborns in Malawi, a high–HIV and –malaria prevalence setting.

## METHODS

### Study Design and Setting

Between January 2013 and February 2014, we undertook a cross-sectional study of mother–newborn pairs at 2 sites in southern Malawi: (i) Queen Elizabeth Central Hospital (QECH), a tertiary referral government hospital in Blantyre covering an urban and peri-urban population of 1.3 million with a high HIV prevalence (17.8% among adults) [21] and low malaria transmission; (ii) Chikwawa District Hospital, covering a rural district with high year-round transmission of *Plasmodium falciparum* [22] and a 13.4% HIV prevalence [21]. Antiretroviral treatment (ART) coverage among known HIV-infected adults and pregnant women in Malawi was around 80% [23]. Sentinel severe acute respiratory illness (SARI) surveillance was undertaken in Blantyre [24] but not in Chikwawa during the study period. There is no national influenza vaccination program.

### Study Participants

Pregnant women aged ≥18 years presenting for delivery were enrolled in the labor ward at the 2 hospitals (see Supplementary Figure 1 for eligibility criteria). Recruitment in Chikwawa was conducted as part of a randomized controlled trial (ISTp) that compared the effectiveness of scheduled intermittent screening with rapid diagnostic tests (RDT) and treatment with dihydroartemisinin-pyrimethamine and intermittent preventative therapy with sulfadoxine-pyrimethamine to prevent malaria during pregnancy in HIV-negative mothers (ISRCTN Registry ISRCTN69800930) [25]. HIV-infected mothers were excluded from the trial.

### Study Procedures

#### Sample Collection and Processing 

Maternal venous blood was collected within 12 hours of delivery. Cord blood was collected at delivery. Sera (Blantyre) and heparinized plasma (Chikwawa) were separated and stored at –80°C until analysis.

HIV status was assessed using sequential rapid tests (Alere Determine HIV-1/2 and Trinity Biotech Uni-Gold HIV) [26]. RDT for malaria (Paracheck Pf, Orchid Biomedical Systems, Goa, India) was performed as per the manufacturer’s instructions.

HAI assay was undertaken at the National Institute for Communicable Diseases (NICD) in Johannesburg, South Africa. Patient sera and plasma were treated with receptor-destroying enzyme (Denka Seiken RDE II), then heat-inactivated and diluted. Serial dilutions of sera and plasma were incubated with equal volumes of reference antigens A/California/7/2009 (A(H1N1)pdm09), A/Victoria/361/2011 (A(H3N2)), B/Brisbane/60/2008 (B/Victoria-lineage), and B/Wisconsin/1/2010 (B/Yamagata-lineage; vaccine strains for Southern and Northern Hemispheres during study period; 2012 VIDRL-WHO influenza virus typing kit: www.influenzacentre.org) at 4 hemagglutinating units each. After 1-hour incubation, an equal volume of 0.5% turkey red blood cells was added and left to settle for 45 minutes. Plates were visually inspected to determine HAI titers. Control reagents were included to monitor for nonspecific agglutination. HAI titer was expressed as the reciprocal of the highest serum dilution where heamagglutination was inhibited. HAI titers in sera and plasma were compared in a random subset of Blantyre mother–infant pairs.

After delivery, a standard questionnaire was administered to mothers to collect demographic data, pregnancy and childbirth history, and socioeconomic indicators (including maternal education, asset ownership, access to sanitation and water facilities, and housing materials). Newborn infants were weighed on digital scales; infants weighing <2500 grams were classified as low birth weight. Due to poor recall of last menstrual period at the time of delivery and limited access to prenatal ultrasound, the gestational age of the newborn was assessed using the modified Ballard score [27]. Those with a gestational age <37 weeks were classified as preterm. A socioeconomic index, divided into tertiles, was generated using principal components analysis [28].

Placental biopsies were collected and fixed in 10% neutral buffered formalin, then processed and embedded in paraffin wax. Four-micron-thin sections were stained with hematoxylin and eosin. The slides were read for presence of parasitized erythrocytes and hemozoin pigment and categorized as “active infection” if parasitized erythrocytes were present, “chronic infection” if both parasitized erythrocytes and pigmented macrophages were present, “past infection” if only pigment was present in fibrin, and “no infection” if no parasites or pigment was present [29]. Active and chronic infection was classified as “placental malaria-positive,” whereas past and no infection was classified as “placental malaria-negative.”

### Statistical Analysis

Analysis was stratified by site due to distinct recruitment periods at the 2 sites (Supplementary Figure 2), recruitment of HIV-infected mothers from Blantyre only, and HAI measurement from different blood components (serum in Blantyre and plasma in Chikwawa). Thus, the impact of maternal HIV infection on maternal–fetal influenza antibody transfer was studied within Blantyre mother–infant pairs, whereas the effect of placental malaria was evaluated among Chikwawa mother–infant pairs.

A previous study in Malawi found a 21% and 18% reduction in placental transfer of IgG antibodies to *Streptococcus pneumoniae* by maternal HIV infection and placental malaria, respectively [14]. Assuming that transplacental transfer of influenza antibodies would be attenuated by a similar magnitude as that observed in pneumococcal antibodies, a sample size of 225 mother–infant pairs per site would provide 80% power to detect a ≥20% difference in the proportion of newborns with an HAI titer ≥1:40 to the 4 circulating influenza strains.

Statistical analyses were performed using Stata, version 14.1 (StataCorp LLC, College Station, TX). Baseline characteristics of enrolled mothers and newborns were compared using the X^2^ test for categorical data and the Wilcoxon rank-sum test for continuous data. Maternal and cord HAI titers against the influenza A(H1N1)pdm09, A(H3N2), B/Victoria, and B/Yamagata viruses were logarithmically transformed to calculate geometric mean titers (GMTs) and 95% confidence intervals (CIs). HAI titers <1:10 were assigned a value of 1:5, and those with titers >1:1280 were assigned a titer of 1:2560. The primary end point was the proportion of infants with HAI titers of ≥1:40 (considered seropositive), which is associated with a ≥50% reduction in symptomatic influenza in young healthy adults [30, 31].

Logistic regression was used to estimate the association of maternal HIV infection and placental malaria with infant influenza seropositivity, as well as the role of possible confounding covariates, including maternal age, parity, gestational age (<37 or ≥37 weeks), infant birth weight (<2500 or ≥2500 grams), socioeconomic status, and season of recruitment. Multivariable models were built using stepwise backward elimination of covariates with *P* values >.20. Significant variables were included in the multivariable models for all 4 strains. Maternal HAI titers were considered to be on the causal pathway in the analyses of infant HAI seropositivity; hence they were excluded from the regression models. A 2-sided *P* value of <.05 was considered significant.

Linear regression models were used to investigate the impact of HIV and placental malaria infection on the association between log maternal and cord titers, adjusted for any identified risk factors for infant seropositivity. The slope of the linear regression line (b1) represents the ratio between log cord and maternal HAI titers (cord-maternal ratio), providing an estimate of the efficiency of transplacental maternal antibody transfer.

### Ethics Statement

The study was approved by the University of Malawi College of Medicine Research Ethics Committee, Blantyre, Malawi. The US Centers for Disease Control and Prevention (CDC) reviewed the protocol and relied on the University of Malawi Ethics Committee (CDC IRB#6507). Written informed consent was obtained from all participating women.

## RESULTS

Recruitment took place between January and August 2013 in Chikwawa and between September 2013 and February 2014 in Blantyre (Supplementary Figure 2). Overall, 454 mother–newborn pairs (Blantyre, n = 253; Chikwawa, n = 201) were included in the analysis (Table 1; Supplementary Figure 3). The median maternal age (interquartile range [IQR]) was 22 (19–28) years. Forty percent of women were primigravid. Thirty-seven of 253 (14.6%) Blantyre mothers were HIV positive. Placental malaria was identified in 10 of 214 (4.7%) Blantyre women and 63 of 201 (31.3%) Chikwawa women. Only 1 enrolled mother in Blantyre had HIV and placental malaria co-infection. Three of 243 (1.2%) Blantyre mothers and 38 of 200 (19.1%) Chikwawa mothers had a positive malaria RDT. None of the mothers had received influenza vaccination.

**Table 1.** Demographic and Clinical Characteristics of Enrolled Mothers and Infants

Characteristic	All, No. (%)	Blantyre, No. (%)	Chikhwawa, No. (%)
	n = 454	n = 253	n = 201
Maternal			
Demographics			
Age, median (IQR), y	22 (19–28)	25 (19–31)	20 (17–23)
Area of residence	n = 448	n = 249	n = 199
Urban	142 (31.7)	141 (56.6)	0 (0)
Peri-urban	67 (15.0)	67 (26.9)	0 (0)
Rural	239 (53.3)	41 (16.5)	199 (100)
SES index score (tertiles)	n = 429	n = 229	n = 200
Low	150 (35.0)	17 (7.4)	133 (66.5)
Medium	137 (31.9)	73 (31.9)	64 (32.0)
High	142 (33.1)	139 (60.7)	3 (1.5)
Highest level of schooling	n = 452	n = 252	n = 202
None	33 (7.3)	6 (2.4)	27 (13.5)
Primary	269 (59.5)	119 (47.2)	150 (75.0)
Secondary	131 (29.0)	108 (42.9)	23 (115)
Tertiary	19 (4.2)	19 (7.5)	0 (0)
Primigravidae	183 (40.3)	84 (33.2)	99 (49.3)
Laboratory findings			
HIV-infected	37 (8.2)	37 (14.6)	0 (0)^a^
Placental malaria	73/415 (17.6)	10/214 (4.7)	63 (31.3)
Positive malaria RDT	41/442 (9.3)	3/243 (1.2)	38/200 (19.1)
Newborn			
Male sex	222/440 (49.4)	123/250 (49.2)	99/200 (49.8)
Gestational age^b^	n = 453	n = 251	n = 197
≥37 wk	413 (92.2)	224 (89.2)	189 (95.9)
<37 wk	35 (7.8)	27 (10.6)	8 (4.1)
Birth weight, median (IQR), g	3000 (2740–3200)	3000 (2800–3240)	3000 (2700–3200)
Low birth weight	36/452 (8.0)	23/257 (9.0)	13/200 (6.5)
Head circumference, mean ± SD, cm	33.3 ± 2.1	33.0 ± 2.6	33.7 ± 1.3

Abbreviations: IQR, interquartile range; RDT, rapid diagnostic test; SES socioeconomic status.

^a^HIV-positive women were excluded in the Chikwawa study.

^b^Based on Ballard score.

Among the newborns, 224 (49.4%) were males. The median gestational age of the babies (range) was 39 (30–42) weeks. Thirty-five of 448 (7.8%) newborns with an available Ballard score were preterm. Low birth weight was observed in 36 (7.9%) infants, of whom 17 (48.6%) were preterm.

### Influenza Antibody Titers in Mothers and Infants, by Maternal HIV and Placental Malaria Status

The proportion of mothers and newborns with HAI titers ≥1:40 against the circulating influenza strains and the corresponding GMT by maternal HIV and placental malaria status are shown in Supplementary Tables 1 and 2. Among Blantyre mother–infant pairs, both HIV-infected mothers and their newborns had a lower proportion of seropositivity for influenza A(H1N1)pdm09 and A(H3N2), compared with HIV-uninfected mother–infant pairs (influenza A(H1N1)pdm09: mothers, 24.3% vs 45.4%; *P* = .02; infants, 24.3% vs 50.5%; *P* = .003; influenza A(H3N2): mothers, 37.8% vs 63.9%; *P* = .003; infants, 43.2% vs 64.8%; *P* = .01) (Supplementary Table 1). HIV-infected mothers and their infants also had significantly lower GMTs for HAI antibodies for both influenza A strains (GM ratio for influenza A(H1N1)2009, 0.59; 95% CI, 0.35–0.99; A(H3N2), 0.45, 95% CI, 0.29–0.71). Percent seropositivity among mother–infant pairs was lower for B/Victoria (8.3%–13.5%) and B/Yamagata (11.1%–19.9%), with no difference by HIV status.

Among Chikwawa mother–infant pairs, seropostivity to the 4 different influenza strains varied from 66.7% to 100% among mothers and 66.7% to 98.6% among infants (Supplementary Table 2). Placental malaria was associated with lower maternal seropositivity (78.7 vs 91.1%; *P* = .02) and GMT (121.7 vs 239.2; GM ratio, 0.51; 95% CI, 0.32–0.80) for influenza A(H1N1)pdm09, but this difference was not observed in the infants. Placental malaria status had no impact on maternal and infant seropositivity for the other influenza strains, though mothers with placental malaria and their newborns had lower GMT for B/Victoria compared with those without placental malaria (GMT in mothers, 418.7 vs 663.9; GM ratio, 0.63; 95% CI, 0.37–1.06; GMT in infants, 96.0 vs 150.2; GM ratio, 0.64; 95% CI, 0.41–1.01).

In the 34 Blantyre mother–infant pairs that had HAI assay performed on both sera and plasma, HAI GMT from plasma were generally higher than the corresponding serum values for all 4 viruses tested, but the difference by blood component was significant for B/Victoria (GMT, 46.6 vs 9.3; GM ratio, 5.01; 95% CI, 2.73–9.18) and B/Yamagata (GMT, 233.3 vs 15.2; GM ratio, 15.36; 95% CI, 8.43–27.98) (Supplementary Table 3).

### Factors Associated With Infant Seropositivity

Among Blantyre mother–infant pairs, maternal HIV was associated with a significantly lower proportion of infants with an HAI titer ≥1:40 for influenza A(H1N1)pdm09 (24.3% vs 50.5%; adjusted odds ratio [aOR], 0.34; 95% CI, 0.15–0.79) and A(H3N2) (43.2% vs 64.8%; OR, 0.43; 95% CI, 0.21–0.89) (Table 2). There was no association between maternal HIV and infant seropositivity for the influenza B strains. Younger maternal age (<25 years) was associated with infant seropositivity for influenza A(H1N1)pdm09 (aOR, 2.61; 95% CI, 1.54–4.45) and influenza B/Yamagata (aOR, 1.83; 95% CI, 0.97–3.79). Additionally, January to April recruitment was associated with infant seropositivity for influenza A(H1N1)pdm09 (aOR, 2.3; 95% CI, 1.34–4.20) and influenza B/Victoria (aOR, 2.40; 95% CI, 1.13–5.10).

**Table 2.** Association of Maternal HIV Status and Other Factors With Infant HAI Titers ≥1:40 in Blantyre

	HAI Titer ≥1:40, No. (%)	Univariable^a^		Multivariable^b^	
		OR (95% CI)	*P* Value	aOR (95% CI)	*P* Value
A(H1N1)pdm09					
Maternal age, y					
18–24	71/121 (58.7)	2.57 (1.55–4.27)	<.001	2.61 (1.54–4.45)	<.001
≥25	47/132 (35.6)	1		1	
Maternal HIV status					
Negative	109/216 (50.5)	1		1	
Positive	9/37 (24.3)	0.32 (0.14–0.70)	.005	0.34 (0.15–0.79)	.01
Season of recruitment					
Sep–Dec	70/171 (40.9)	1		1	
Jan–Apr	48/81 (59.3)	2.10 (1.23–3.59)	.007	2.38 (1.35–4.20)	.003
A(H3N2)					
Maternal age, y					
18–24	80/121 (66.1)	1.44 (0.86–2.40)	.16	1.36 (0.81–2.30)	.25
≥25	76/132 (57.6)	1		1	
Maternal HIV status					
Negative	140/216 (64.8)	1		1	
Positive	16/37 (43.2)	0.41 (0.20–0.84)	.02	0.43 (0.21–0.89)	.02
Season of recruitment					
Sep–Dec	104/171 (60.8)	1		1	
Jan–Apr	52/81 (64.2)	1.16 (0.67–2.00)	.61	1.18 (0.67–2.06)	.56
B/Victoria					
Maternal age, y					
18–24	16/121 (13.2)	1.10 (0.53–2.32)	.79	1.17 (0.55–2.50)	.68
≥25	15/132 (12.1)	1		1	
Maternal HIV status					
Negative	27/216 (12.5)	1		1	
Positive	5/37 (13.5)	1.09 (0.39–3.05)	.86	1.12 (0.39–3.18)	.84
Season of recruitment					
Sep–Dec	16/171 (9.4)	1		1	
Jan–Apr	16/81 (19.8)	2.38 (1.13–5.05)	.02	2.40 (1.13–5.10)	.02
B/Yamagata					
Maternal age, y					
18–24	30/121 (24.8)	1.85 (0.98–3.47)		1.84 (0.97–3.49)	.06
≥25	20/132 (15.2)	1		1	
Maternal HIV status					
Negative	43/216 (19.9)	1		1	
Positive	7/37 (18.9)	0.94 (0.39–2.28)	.89	1.08 (0.44–2.69)	.86
Season of recruitment					
Sep–Dec	30/171 (17.5)	1		1	
Jan–Apr	19/81 (23.5)	1.44 (0.75–2.75)	.27	1.49 (0.77–2.86)	.23

Abbreviations: aOR, adjusted odds ratio; CI, confidence interval; HAI, hemagglutination inhibition.

^a^Potential confounders assessed: Maternal: age, socioeconomic status index, parity. Infant: sex, gestational age, birth weight. Other: season of recruitment.

^b^Multivariable logistic regression: Maternal age, maternal HIV status, and season of recruitment.

Placental malaria had no association with infant seropositivity for any of the influenza strains in Chikwawa (Table 3). Younger maternal age was associated with infant seropositivity against influenza A(H3N2) (71.3 vs 51.4%; aOR, 2.50; 95% CI, 1.18–5.27). Conversely, older maternal age was associated with infant seropositivity for influenza B/Victoria (82.2 vs 94.7%; aOR, 4.29; 95% CI, 0.96–19.16). Recruitment between May and August was associated with increased odds of infant seropositivity for influenza B/Victoria.

**Table 3.** Association of Placental Malaria and Other Factors With Infant HAI Titers ≥1:40 in Chikwawa

	HAI Titer ≥1:40, No. (%)	Univariable^a^		Multivariable^b^	
		OR (95% CI)	*P* Value	aOR (95% CI)	*P* Value
A(H1N1)pdm09					
Maternal age, y					
18–24	135/158 (85.4)	0.50 (0.14–1.77)	.29	0.53 (0.15–1.92)	.34
≥25	35/38 (92.1)	1		1	
Placental malaria					
Negative	119/135 (88.2)	1		1	
Positive	51/61 (83.6)	0.69 (0.29–1.61)	.39	0.73 (0.31–1.75)	.48
Season of recruitment					
Jan–Apr	126/145 (86.9)	1.05 (0.42–2.68)	.91	1.06 (0.41–2.72)	.90
May–Aug	44 (86.3)	1		1	
A(H3N2)					
Maternal age, y					
18–24	114/160 (71.3)	2.35 (1.13–4.87)	.02	2.50 (1.18–5.27)	.02
≥25	19/37 (51.4)	1		1	
Placental malaria					
Negative	91/134 (67.9)	1		1	
Positive	42/63 (66.7)	0.95 (0.50–1.78)	.86	0.80 (0.41–1.55)	.51
Season of recruitment					
Jan–Apr	101/147 (68.7)	1.24 (0.63–2.42)	.61	1.32 (0.66–2.64)	.43
May–Aug	32/50 (64.0)	1		1	
B/Victoria					
Maternal age, y					
18–24	134/163 (82.2)	1		1	
≥25	36/38 (94.7)	3.90 (0.89–17.10)	.07	4.29 (0.96–19.16)	.06
Placental malaria					
Negative	118/138 (85.5)	1		1	
Positive	52/63 (82.5)	0.80 (0.36–1.79)	.59	1.01 (0.44–2.33)	.98
Season of recruitment					
Jan–Apr	121/150 (80.7)	1		1	
May–Aug	49/51 (96.1)	5.87 (1.35–25.56)	.02	6.27 (1.43–27.48)	.02
B/Yamagata					
Maternal age, y					
18–24	161/163 (98.8)	2.18 (0.19–24.6)	.53	2.39 (0.19–29.40)	.50
≥25	37/38 (97.4)	1		1	
Placental malaria					
Negative	136/138 (98.6)	1		1	
Positive	62/63 (98.4)	0.93 (0.08–10.40)	.95	0.74 (0.06–9.16)	.81
Season of recruitment					
Jan–Apr	148/150 (98.7)	1.38 (0.13–16.67)	.75	1.61 (0.14–18.89)	.70
May–Aug	50/51 (98.0)	1			

Abbreviations: aOR, adjusted odds ratio; CI, confidence interval; HAI, hemagglutination inhibition.

^a^Potential confounders assessed: Maternal: age, socioeconomic status index, parity. Infant: sex, gestational age, birth weight. Other: season of recruitment.

^b^Multivariable logistic regression. Maternal age, placental malaria, and season of recruitment were included in all multivariable models.

### Transplacental Transfer of Maternal Influenza Antibodies

**Figure 1.**
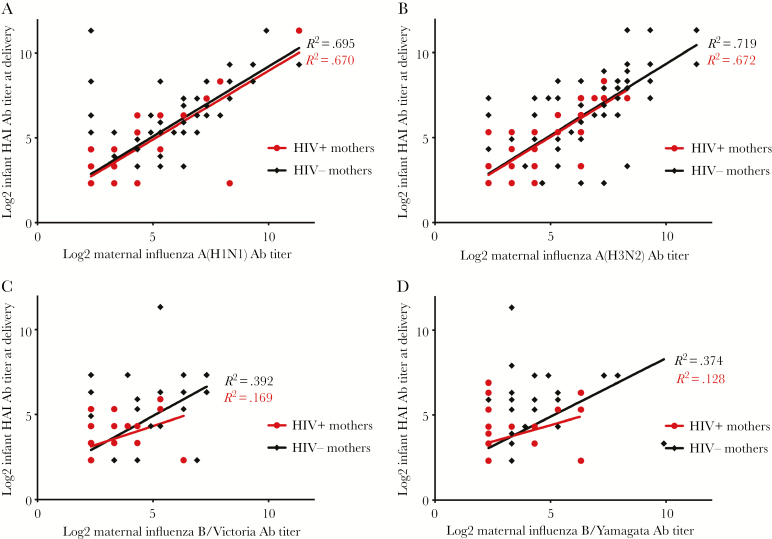
Relationship between maternal and newborn HAI titers among Blantyre mother–infant pairs by maternal HIV status. Abbreviations: Ab, antibody; HAI, hemagglutination inhibition.

**Figure 2.**
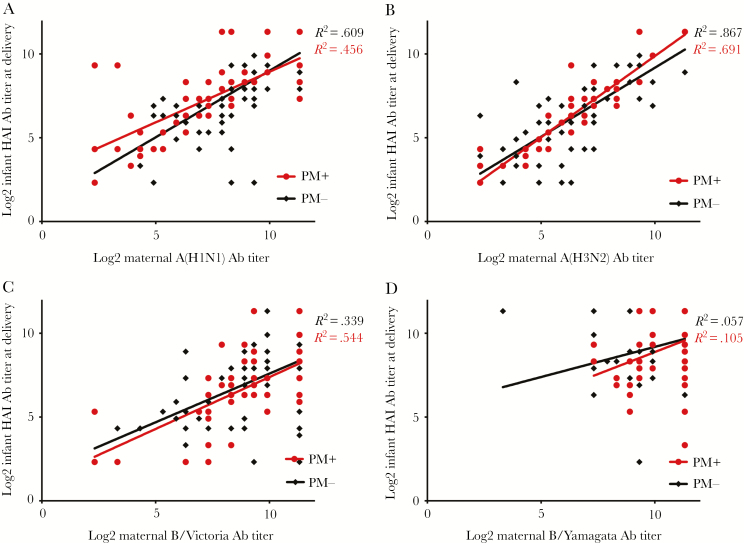
Relationship between maternal and newborn HAI titers among Chikwawa mother–infant pairs by placental malaria status. Abbreviations: Ab, antibody; HAI, hemagglutination inhibition; PM, placental malaria.

Figures 1 and 2 illustrate the relationship between maternal and cord blood HAI titers by maternal HIV and placental malaria status, respectively. Among Blantyre mother–infant pairs, a strong positive linear relationship between maternal and cord HAI titers was observed for influenza A(H1N1)pdm09 (HIV-uninfected mothers: b1 = .825; *R*^2^ = .695; HIV-infected mothers: b1 = .810; *R*^2^ = .670) and A(H3N2) (HIV-uninfected mothers: b1 = .841; *R*^2^ = .719; HIV-infected mothers: b1 = .834; *R*^2^ = .672), suggesting efficient transplacental transfer (Figure 1; Supplementary Table 4). In contrast, poor linear association between maternal and infant titers was observed for B/Victoria and B/Yamagata. The slopes of the regression lines between HIV-infected and HIV-uninfected mother–infant pairs were not significantly different for all 4 strains, suggesting that maternal HIV had no impact on transplacental transfer (Supplementary Table 4).

Among Chikwawa mother–infant pairs, some linear association between maternal and cord HAI titers against influenza A(H1N1)pdm09 was observed (placental malaria-positive mothers, *R*^2^ = .456; placental malaria-negative mothers, *R*^2^ = .609) and against A(H3N2) (placental malaria-positive mothers, *R*^2^ = .867; placental malaria-negative mothers, *R*^2^ = .691), but not for B/Victoria and B/Yamagata (Figure 2). The slope of the regression line differed by placental malaria status for influenza A(H1N1)pdm09 (placental malaria-positive vs placental malaria-negative: b1 = .604 vs .796; *P* = .05) and A(H3N2) (placental malaria-positive vs placental malaria-positive: b1 = .970 vs .823; *P* = .06) (Supplementary Table 4). There was no difference in the slopes of the regression lines for B/Victoria and B/Yamagata by placental malaria status.

## Discussion

HIV-infected mothers and their newborns had lower percent seropositivity and HAI titers to influenza A, but not B viruses, compared with HIV-negative mother–infant pairs. Maternal HIV infection did not affect transplacental antibody transfer. In contrast, placental malaria had no consistent impact on maternal and newborn influenza antibody levels or transplacental transfer. Season of recruitment and young maternal age were also independently associated with infant seropositivity.

There are 2 possible explanations for the lower antibody responses observed in HIV-infected mothers: (i) HIV-infected mothers generate weaker humoral responses to influenza virus infection than mothers without HIV, and (ii) HIV-infected pregnant women avoid crowded areas to mitigate infection exposure and therefore have a lower probability of influenza infection. Existing evidence supports the former; an influenza vaccine trial in pregnant women found lower antibody responses to all 3 vaccine strains in HIV-infected mothers, compared with HIV-uninfected women [20]. Interestingly, lower HAI titers did not translate to poorer clinical efficacy [9]. Infants of HIV-infected mothers also had lower titers, but insufficient power precluded evaluation of vaccine efficacy [20]. Second, studies have reported a greater burden of symptomatic influenza in adults [17] and pregnant women [18] with HIV infection than those without HIV. Therefore, it seems unlikely that HIV-infected mothers in our study would have experienced fewer influenza infections than HIV-uninfected women. Furthermore, a study exploring the community perceptions of influenza in Malawi did not identify avoidance of crowded places as a known strategy to prevent the spread of influenza [32]. We found no difference in influenza B/Victoria and B/Yamagata seropositivity by maternal HIV status, which we speculate may be due to the comparatively low prevalence of influenza B seropositivity among Blantyre mother–infant pairs.

Malaria infection during pregnancy can affect antibody receptors on the placental surface [33]. Several studies have reported reduced transfer of antibodies against measles [14, 33] and tetanus [13, 16] in the presence of placental malaria, whereas others have not [15]. To our knowledge, the effect of placental malaria on the transplacental transfer of influenza antibodies has not been evaluated. We found no association between placental malaria and infant influenza seropositivity. Additionally, placental malaria had no consistent impact on transplacental transfer of influenza antibodies; it was associated with less efficient transfer of influenza A(H1N1)pdm09 antibodies, but more efficient antibody transfer to A(H3N2). Scott et al. found reduced transplacental transfer of measles antibodies only in mothers with active chronic placental malaria infection [15]. We classified acute and chronic placental malaria as “positive” and those with past placental malaria infection as “negative.” A sensitivity analysis demonstrated no difference in transplacental transfer when placental malaria status was stratified as acute, chronic, past, or no infection, except for those with acute infection for influenza A/California/7/2009 (Supplementary Figure 4). The observed difference in the slope of the regression line is not significant as there was no linear relationship between log maternal and infant titer (*R*^2^ = .033).

Infants with mothers aged <25 years had an approximately 2-fold increased odds of seropositivity against influenza A(H1N1)pdm09 and B/Yamagata in Blantyre, as well as A(H3N2) in Chikwawa, compared with infants of older mothers. Young age was also a predictor of antibody response in several influenza vaccine studies in HIV-infected and HIV-uninfected adults [20, 34]. In HIV-uninfected young individuals, this may be due to better thymic function [20]. In HIV-infected adults, poorer antibody response with increasing age may be associated with longer duration of HIV infection or, with the former national HIV treatment guidelines that recommended commencement of ART at a lower CD4+ threshold, a longer duration of HIV-mediated immune dysregulation before ART initiation.

Influenza seropositivity was high among mothers and their newborns to the influenza A viruses in Blantyre and to all 4 circulating influenza viruses in Chikwawa. In the context of no influenza vaccination coupled with evidence of influenza virus circulation, it is reasonable to assume that detected antibodies reflect exposure to natural influenza infection. Seropositivity and GMTs varied substantially between Blantyre and Chikwawa mother–infant pairs, particularly for influenza B viruses. Although information on circulating influenza strains was only available in Blantyre, we postulate that this variability is partly due to exposure to different circulating strains from distinct recruitment periods. Moreover, the different blood components on which HAI assays were performed is likely a contributory factor. Analysis of paired plasma and sera in our subset of Blantyre samples demonstrated significantly higher HAI titers from plasma compared with sera for the influenza B viruses, which has been previously reported [35].

Our study had several limitations. First, because pregnant women were recruited at the time of delivery, we were unable to determine the maternal history of influenza infection and could not definitively conclude whether low maternal antibody levels were due to poor response to natural infection or absence of exposure. Second, CD4+ cell count and ART status of HIV-infected mothers were not captured. Thus we were unable to assess the effect of varying degrees of immunosuppression on antibody responses in HIV-infected mothers and infants. Third, analysis was stratified by site as a result of the differences in recruitment time periods, blood components used for HAI titer, and eligibility criteria (HIV-infected women excluded in Chikwawa), thus precluding evaluation of the effect of maternal HIV and placental malaria co-infection and their potential interaction on transplacental influenza antibody transfer, as only 1 mother from Blantyre had dual infection. Misclassification of prematurity is possible, as the modified Ballard score is prone to inter-rater variability [36] and tends to overestimate gestational age [37]. Lastly, we chose a cord HAI titer of ≥1:40 as a correlate for protection [30, 31]. Some have argued that higher HAI titers (eg, 1:110) may be required to provide protection against influenza in children due to an immature cellular immune system and a lack of immunological memory [38]. Furthermore, a recent study suggested that microneutralization (MN) titers may be better predictors of protection compared with HAI [39].

In summary, infants of HIV-infected mothers were more likely to be seronegative and have lower antibody titers to influenza A viruses, which we postulate is due to an attenuated maternal antibody response to natural influenza infection rather than reduced efficiency of placental transfer. In contrast, placental malaria had no consistent impact with infant influenza seropositivity or transplacental transfer. These findings suggest that influenza vaccines targeting pregnant women may have variable efficacy in sub-Saharan Africa, where up to 35% of women of childbearing age may be HIV infected [40]. Adjuvanted preparations or higher doses to boost maternal antibodies may be required to optimize protection for HIV-infected pregnant women and their infants, which will impact the cost-effectiveness of maternal influenza immunization interventions. Further studies are needed to define the best correlate of protection against influenza disease in HIV-infected persons and clarify whether the attenuated antibody response observed in infants of HIV-infected women results in reduced clinical protection, if it is compounded by dual maternal HIV and placental malaria infection, and whether ART improves influenza antibody responses in HIV-infected mothers and infants.

## Supplementary Data

Supplementary materials are available at *Open Forum Infectious Diseases* online. Consisting of data provided by the authors to benefit the reader, the posted materials are not copyedited and are the sole responsibility of the authors, so questions or comments should be addressed to the corresponding author.

ofz383_suppl_supplementary_material
