# Supplementary material for: Impact of Maternal HIV Infection and Placental Malaria on the Transplacental Transfer of Influenza Antibodies in Mother–Infant Pairs in Malawi, 2013–2014
Source: Open Forum Infect Dis. 2019 Aug 28;6(10):ofz383. doi: 10.1093/ofid/ofz383 (PMC6785697; doi:10.1093/ofid/ofz383)
Supplement: ofz383_suppl_supplementary_material [file ofz383_suppl_supplementary_material.docx]

| **Inclusion criteria** |
| --- |
| - Aged 18 years and over - >30 weeks’ gestation - Able to give informed consent |
| **Exclusion criteria** |
| - Multiple gestation pregnancies - Influenza vaccination in the past year - Fetal death in utero or intrapartum death of the newborn - History of chronic liver or renal disease, heart failure, or those receiving immunosuppressive therapy |

**Supplementary Figure 1. Eligibility criteria for study enrolment.**

**
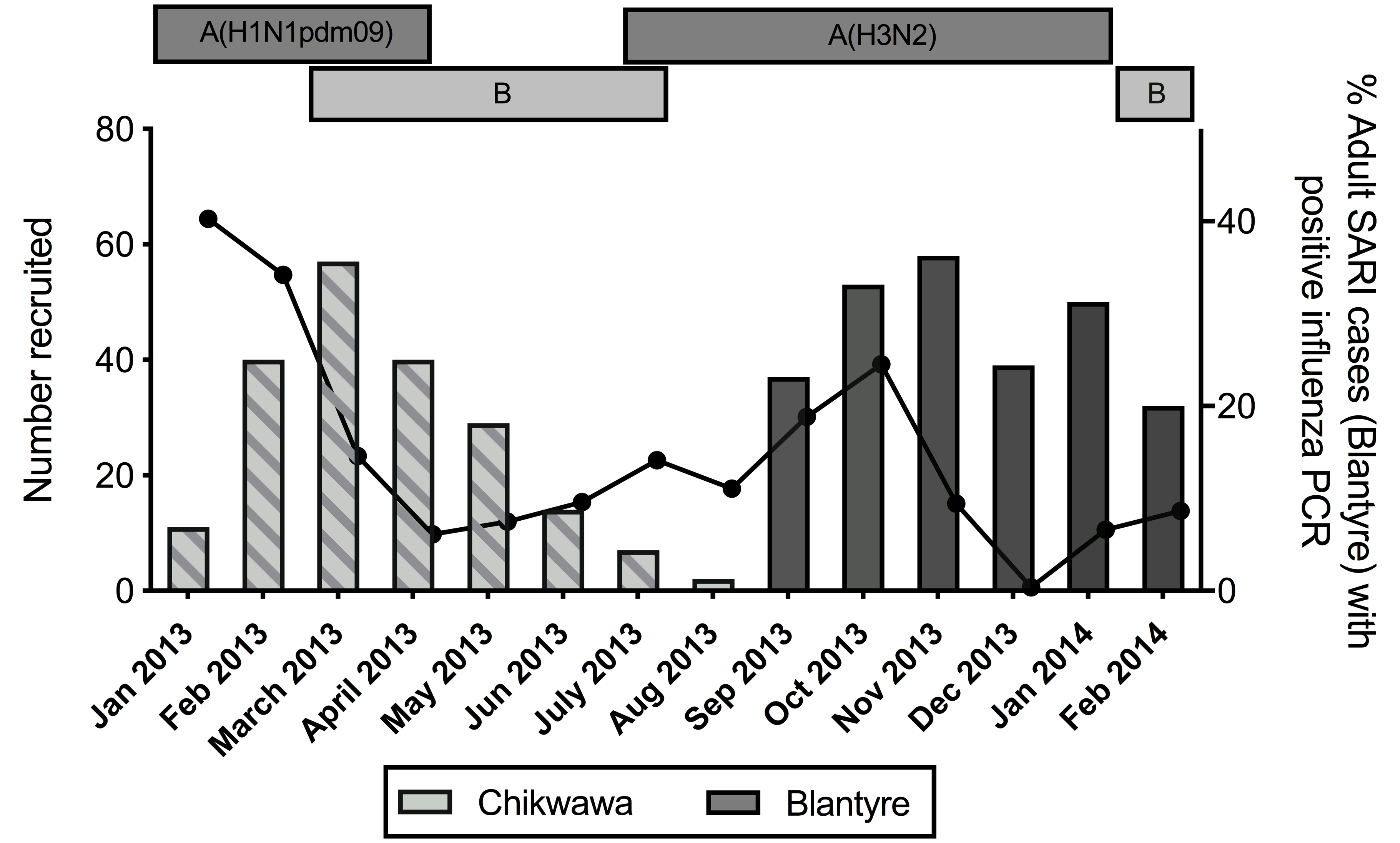
**

**Supplementary Figure 2. Timeline of patient recruitment in Chikwawa and Blantyre, the percentage of influenza-PCR positive SARI cases, and circulating influenza virus type and subtype, from sentinel SARI surveillance at the Queen Elizabeth Central Hospital, Blantyre, January 2013 to February 2014.**

Abbreviations: PCR, polymerase chain reaction; SARI, severe acute respiratory infection

**
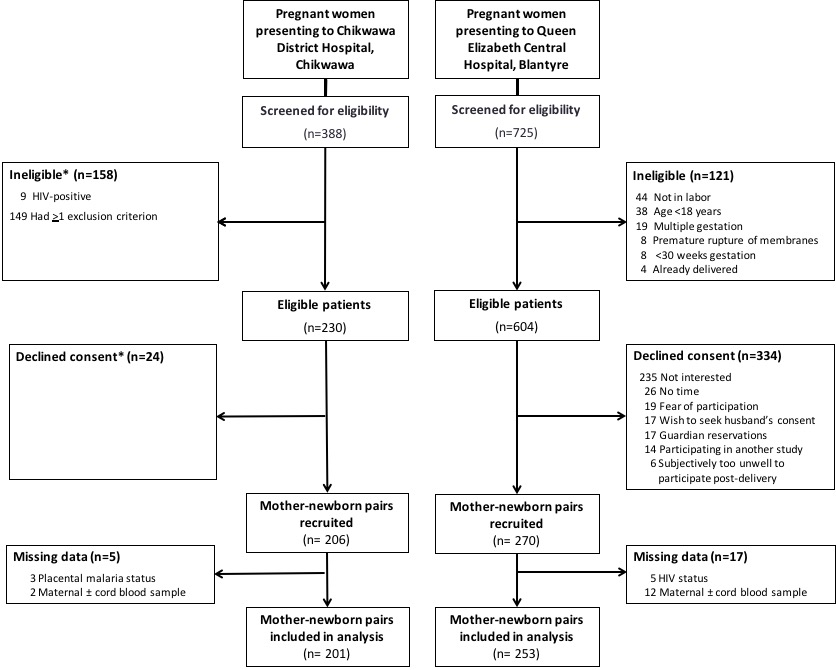
Supplementary Figure 3. Recruitment flow diagram for Chikwawa and Blantyre sites, Malawi, January 2013 to February 2014.**

*Breakdown of reasons for ineligibility and decline of consent not recorded at Chikwawa site.

**Supplementary Table 1. Influenza antibody titers in mothers and infants by HIV status in Blantyre**

|  |  |  | **HIV-positive** | | |  | **HIV-negative** | | |  |  |
| --- | --- | --- | --- | --- | --- | --- | --- | --- | --- | --- | --- |
|  |  | | N | HAI titre >1:40 (%) | GMT |  | N | % with HAI titre >1:40 | GMT | GM ratio | p-value |
| **Mothers** |  | |  |  |  |  |  |  |  |  |  |
| A/California/7/2009 ((H1N1) | | | 37 | 24.3 | 14.4 (8.7-24.1) |  | 216 | 45.4 | 24.4 (20.3-29.4) | 0.59 (0.35-0.99) | 0.04 |
| A/Victoria/361/2011 (H3N2) | | | 37 | 37.8 | 20.2 (12.9-31.7) |  | 216 | 63.9 | 44.5 (36.7-54.0) | 0.45 (0.29-0.71) | 0.001 |
| B/Brisbane/60/2008 (Victoria) | | | 36 | 8.3 | 7.6 (5.9-9.8) |  | 218 | 9.2 | 8.5 (7.8-9.5) | 0.89 (0.69-1.15) | 0.38 |
| B/Wisconsin/1/2010 (Yamagata) | | | 36 | 11.1 | 7.9 (5.8-10.8) |  | 216 | 12.2 | 10.0 (8.9-11.2) | 0.80 (0.58-1.08) | 0.15 |
| **Newborns** |  | |  |  |  |  |  |  |  |  |  |
| A/California/7/2009 ((H1N1) | | | 37 | 24.3 | 15.7 (9.4-26.0) |  | 216 | 50.5 | 27.1 (22.5-32.6) | 0.58 (0.35-0.96) | 0.03 |
| A/Victoria/361/2011 (H3N2) | | | 37 | 43.2 | 22.4 (14.2-35.3) |  | 216 | 64.8 | 46.2 (38.1-55.9) | 0.48 (0.31-0.76) | 0.002 |
| B/Brisbane/60/2008 (Victoria) | | | 37 | 13.5 | 10.5 (8.0-13.8) |  | 216 | 12.5 | 11.2 (9.9-12.7) | 0.94 (0.71-1.24) | 0.65 |
| B/Wisconsin/1/2010 (Yamagata) | | | 37 | 18.9 | 12.6 (9.1-17.4) |  | 216 | 19.9 | 13.3 (11.6-15.2) | 0.94 (0.68-1.31) | 0.73 |

Abbreviations: HIV, human immunodeficiency virus; HAI, haemagglutination inhibition; GMT, geometric mean titer.

**Supplementary Table 2. Influenza antibody titers in mothers and infants by placental malaria status in Chikwawa**

|  |  |  | **Placental malaria** | | |  | **No placental malaria** | | |  |  |
| --- | --- | --- | --- | --- | --- | --- | --- | --- | --- | --- | --- |
|  |  | | N | % with HAI titre >1:40 | GMT (95% CI) |  | N | % with HAI titre >1:40 | GMT (95% CI) | GM ratio | p-value |
| **Mothers** |  | |  |  |  |  |  |  |  |  |  |
| A/California/7/2009 (H1N1) | | | 61 | 78.7 | 121.7 (77.0-192.4) |  | 135 | 91.1 | 239.2 (190.5-300.4) | 0.51 (0.32-0.80) | 0.004 |
| A/Victoria/361/2011 (H3N2) | | | 63 | 69.8 | 75.4 (48.7-116.8) |  | 135 | 66.7 | 68.0 (53.1-87.2) | 1.11 (0.72-1.72) | 0.46 |
| B/Brisbane/60/2008 (Victoria) | | | 63 | 88.9 | 418.7 (248.4-705.6) |  | 138 | 95.6 | 663.9 (520.0-847.6) | 0.63 (0.37-1.06) | 0.08 |
| B/Wisconsin/1/2010 (Yamagata) | | | 63 | 100.0 | 1557.8 (1218.1-1992.2) |  | 138 | 99.3 | 1593.4 (1376.0-1845.2) | 0.98 (0.76-1.25) | 0.86 |
| **Newborns** |  | |  |  |  |  |  |  |  |  |  |
| A/California/7/2009 ((H1N1) | | | 61 | 83.6 | 135.9 (89.5-206.2) |  | 135 | 88.2 | 158.4 (126.0-199.3) | 0.86 (0.56-1.30) | 0.47 |
| A/Victoria/361/2011 (H3N2) | | | 63 | 66.7 | 74.7 (47.8-116.8) |  | 134 | 67.9 | 61.0 (47.9-77.7) | 1.23 (0.78-1.92) | 0.37 |
| B/Brisbane/60/2008 (Victoria) | | | 63 | 82.5 | 96.0 (60.8-151.7) |  | 138 | 85.5 | 150.2 (117.7-191.6) | 0.64 (0.40-1.01) | 0.06 |
| B/Wisconsin/1/2010 (Yamagata) | | | 63 | 98.4 | 587.3 (397.8-867.0) |  | 138 | 98.6 | 684.7 (549.3-853.6) | 0.86 (0.58-1.27) | 0.44 |

Abbreviations: HAI, haemagglutination inhibition; GMT, geometric mean titer.

**Supplementary Table 3. Comparison of HAI titers for 34 paired serum and plasma samples from a subset of Blantyre mothers and infants (n=34), Malawi, January 2013 and February 2014**

| **Influenza virus type/subtype** | **GMT** | | **GMT ratio** | **p-value** |
| --- | --- | --- | --- | --- |
|  | **Serum** | **Plasma** |  |  |
| A/California/7/2009 (H1N1) | 35.4 (20.6-60.9) | 62.0 (30.8-124.3) | 1.75 (0.87-3.51) | 0.11 |
| A/Victoria/361/2011 (H3N2) | 36.9 (22.2-61.2) | 46.6 (28.7-121.0) | 1.26 (0.62-2.60) | 0.52 |
| B/Brisbane/60/2008 (Victoria-lineage) | 9.3 (6.7-12.9) | 46.6 (25.4-85.5) | 5.01 (2.73-9.18) | <0.001 |
| B/Wisconsin/1/2010 (Yamagata-lineage) | 15.2 (9.8-23.6) | 233.3 (128.1-424.9) | 15.36 (8.43-27.98) | <0.001 |

**Supplementary Table 4. Slope of linear regression between log2 transformed maternal and infant HAI titers for circulating influenza strains**

| **Blantyre mother-infant pairs** | | | |
| --- | --- | --- | --- |
| **Influenza type/subtype** | **HIV-infected** | **HIV-uninfected** | **p-value** |
| **A(H1N1)** | 0.810 (0.615-1.006) | 0.825 (0.751-0.898) | 0.88 |
| **A(H3N2)** | 0.834 (0.634-1.034) | 0.841 (0.770-0.911) | 0.95 |
| **B/Victoria** | 0.447 (0.101-0.792) | 0.747 (0.622-0.873) | 0.10 |
| **B/Yamagata** | 0.385 (0.035-0.735) | 0.689 (0.568-0.809) | 0.07 |
| **Chikwawa mother-infant pairs** | | | |
| **Influenza type/subtype** | **Placental malaria** | **No placental malaria** | **p-value** |
| **A(H1N1)** | 0.604 (0.431-0.778) | 0.796 (0685-0.906) | 0.05 |
| **A(H3N2)** | 0.970 (0.873-1.067) | 0.823 (0.727-0.919) | 0.06 |
| **B/Victoria** | 0.619 (0.474-0.764) | 0.581 (0.443-0.719) | 0.72 |
| **B/Yamagata** | 0.526 (0.132-0.920) | 0.360 (0.112-0.607) | 0.48 |

| 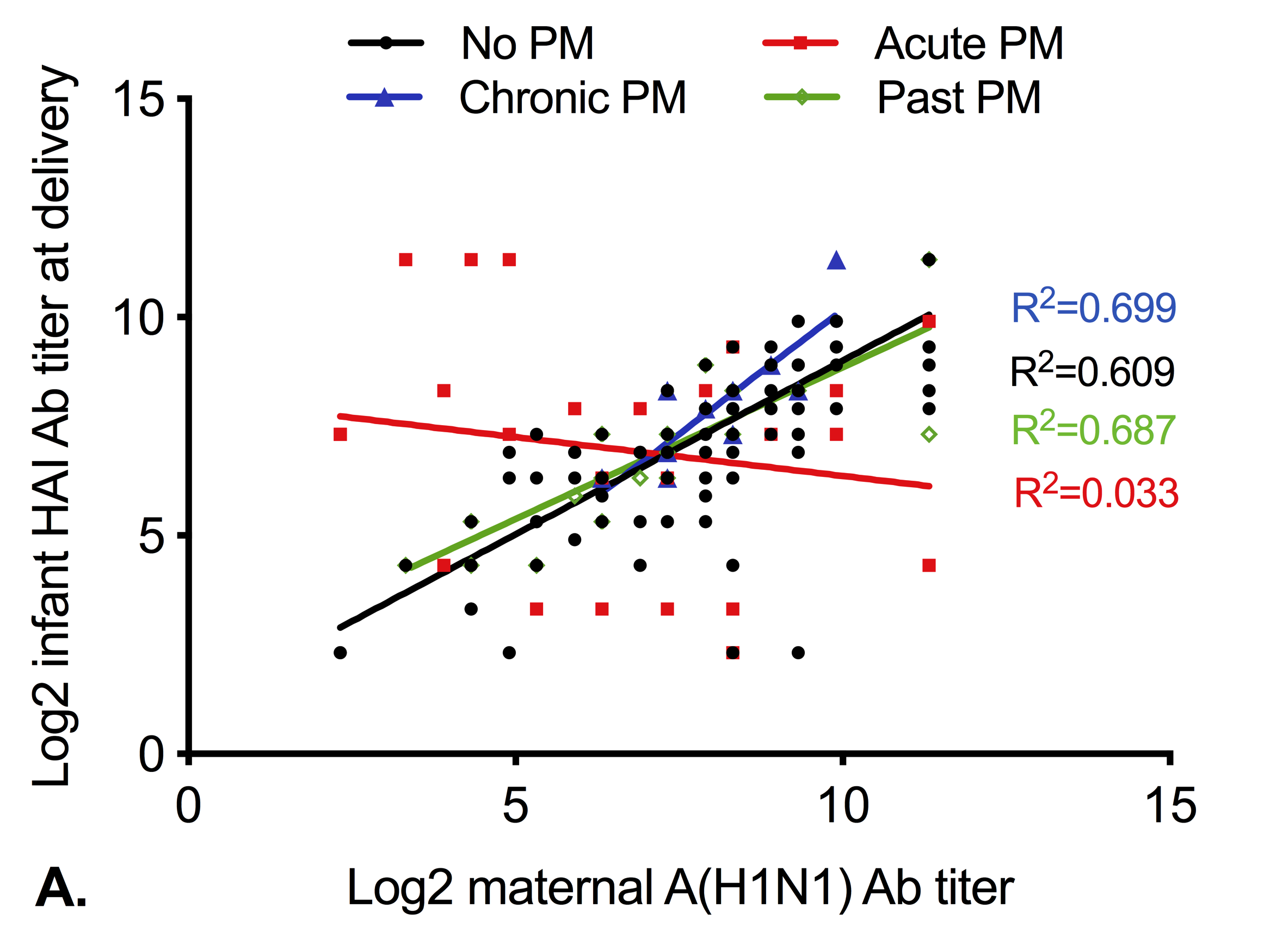 | **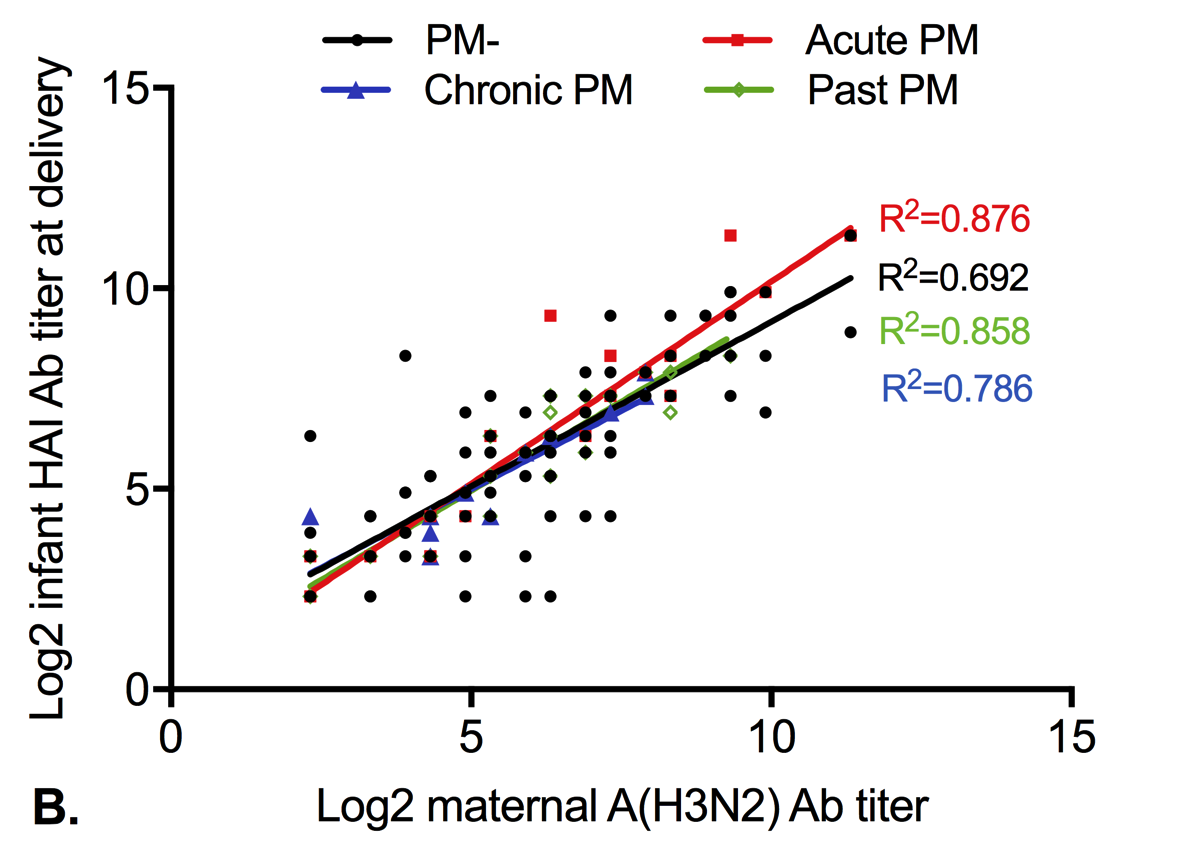** |
| --- | --- |
| **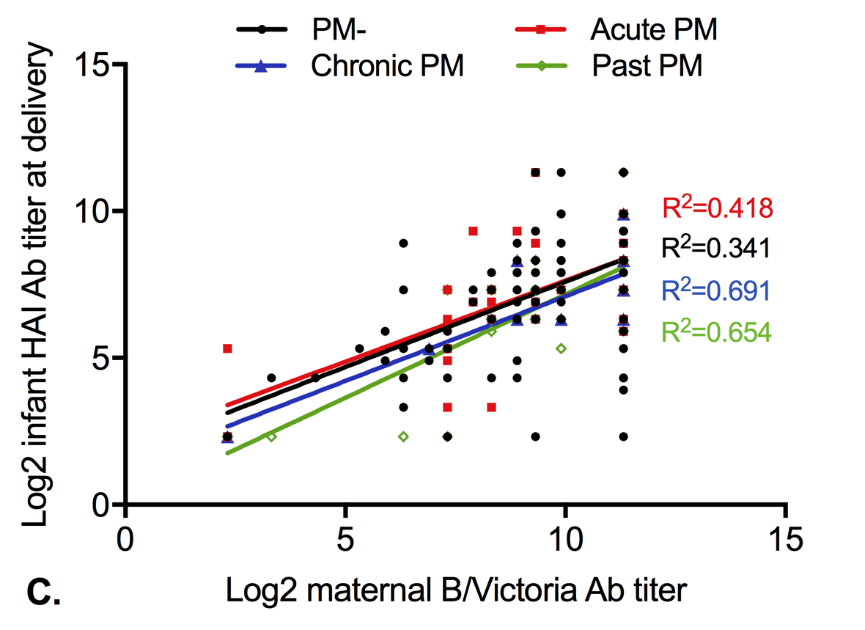** | **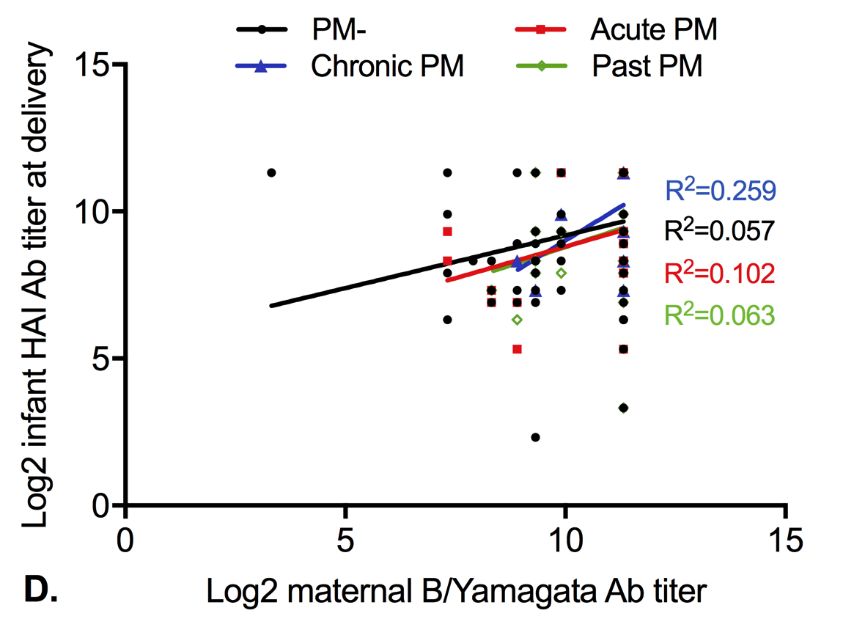** |

**Supplementary Figure 4. Relationship between maternal and newborn HAI titers among Chikwawa mother-infant pairs, by placental malaria status (stratified as acute, chronic, past, or no infection)**
